# What’s in a name? A discussion on the definition of natural and unnatural causes of death

**DOI:** 10.1186/s13010-022-00125-1

**Published:** 2022-11-23

**Authors:** Cécile M. Woudenberg-van den Broek, Koos van der Velden, Wilma L. J. M. Duijst-Heesters

**Affiliations:** 1 Department of Criminal Law and Criminology, Faculty of Law, Maastricht University, P.O. Box 616, 6200 MD Maastricht, The Netherlands; 2 Primary and Community Care, Radboud Institute for Health Sciences, Geert Grooteplein 27, 6525 EZ Nijmegen, The Netherlands

**Keywords:** Forensic medicine, Cause of death, Unnatural death, Natural death, Netherlands, Belgium, Germany, England

## Abstract

When considering the manner of death, two categories can be distinguished, namely natural death and unnatural death. Though most physicians think that the distinction between the two is evident, this is not the case.

When comparing the Netherlands, Belgium, England and Germany it is noticed that the terms natural and unnatural might be used in law but are not defined by law. In practice, the term unnatural death is used when there is an external cause of death, but even that turns out to not be sufficient in making an obvious difference between the two terms. Different countries may even label the same death differently. A, at times philosophical and semantic, discussion shows that when it comes to causes of death a very large grey area exists between natural and unnatural causes of death. The Netherlands, Belgium and Germany even have the possibility to label a death as natural (or unnatural) without actually knowing the cause of death.

In conclusion, we recommend a new system in which the circumstances surrounding a death are properly investigated. This should lead to a report to an independent legal expert, who is able to decide if and what conclusion can be drawn, from a judicial and a public point of view, thereby, making the distinction and the use of the terms natural and unnatural/nonnatural obsolete.

## Introduction

According to article 2 of the European Convention on Human Rights [1], every country needs to have a system to investigate a death. The system needs to comply with several points: being capable of establishing the circumstances surrounding a death, the cause and manner of death, holding accountable those who are at fault and providing appropriate redress to the victim. Every country has its own system, where at some point or other a decision needs to be made whether a death is natural. The Netherlands has a dichotomous system, with natural versus unnatural death. England and Wales also have a dichotomous system, with natural and unnatural cause of death. In Belgian law, the terms ‘natural’, ‘violent’, ‘suspicious’ or ‘undefined cause of death’ are applied. In Germany, the terms used are ‘natural’, ‘unnatural’ and ‘undefined’ death.

A cause of death is seen as the disease or trauma that directly caused the death. A manner of death is the determination of the death as natural or unnatural. The distinction between the two is relevant in all jurisdictions. In this article we discuss the manner of death. The decision whether a death is natural, or not, is of major importance. The conclusion is from a medical point of view, but is then used in a judicial system. From a judicial point of view, it is relevant whether a death is accidental, preventable, blameworthy or potentially criminal.

When physicians and scientists communicate with each other or with the relevant authorities on the subject of death, they use the terms ‘natural and unnatural death’, death by ‘natural or unnatural causes’ or closely related terms. The same goes for physicians and scientists publishing articles on the matter; one assumes that they are all referring to the same concept. But are they really? Though guidelines exist, they vary between medical professions and they give room for discussion within the different medical professions [2].

In this article, we explore the field of natural and unnatural/non-natural death and show that the distinction between the two might be less obvious than presumed, though the discussion may appear philosophical or even semantic at times. Problems may arise if we compare ‘facts’ for which different professionals and different countries apply a different meaning or understanding. Before we can embark on this discussion, we should take a closer look at the various systems used in The Netherlands, Belgium, England and Germany [3].

## Systems

The decision if a death is natural or unnatural is made by either a medical professional (medical system) or a legal professional (coroner or legal system). The Netherlands, Belgium and Germany have a medical system. England has a legal system. Even if at first glance the difference might not give discrepancies in the conclusion of natural or unnatural death, in practice these discrepancies do occur.

Though ultimately the result of an investigation into a death seems to be whether or not it was natural. In the Netherlands, Belgium and Germany the distinction between natural or unnatural death is the first decision to be made, not the decision about the cause of death. Reasons to investigate a death any further are solely based on whether or not a third party might be involved in the death. If this is not the case, or if there are no facts and circumstances that would point to a third party being involved in the death, the prosecutor will see no reason to investigate a case any further, even if the cause of death is not known. In England and Wales on the other hand, the focus is primarily on the cause of death. The decision of whether a death is natural or unnatural is secondary. So, while in England there will be an ultimate conclusion on natural or unnatural death, the most relevant and important question around which the investigation is built, is ‘How did a person come by their death?’. One could wonder how one arrives at the conclusion of natural (or unnatural) death in the Netherlands, Belgium and Germany without knowing what has caused the death in the first place.

As an example, we give the following case. A person dies after abdominal surgery, where a piece of his intestines is removed. Shortly after the operation, the person dies. The different countries would act as follows.

- In the Netherlands, the surgeon would most probably assume that a leakage occurred between the two reattached ends and that the person died from a known complication of such a procedure. A medical complication is seen as a natural cause of death.
- In Germany and Belgium, the surgeon would most definitely assume a natural cause of death and will only mention the possible complication when the family asks questions.
- In England and Wales, the surgeon will report the death to a coroner, because regardless of the fact that this might be a well-known complication followed by death, it is a reportable death and therefore an unnatural cause of death.

## The degree of certainty of the conclusion

In each country, the conclusion of a natural (cause of) death is taken with a degree of (un)certainty. The degree of certainty depends on the amount of investigation done. In the Netherlands, a physician needs to be 'convinced' of a natural death before signing the appropriate forms. This conviction can be a well-reasoned conclusion based on knowledge about and on investigation into the facts and circumstances leading to the death, combined with a thorough external post-mortem examination [4]. It can also be a personal belief not based on any research or investigation whatsoever. For example, a 35-year-old man found deceased in his kitchen at home. His own general practitioner can come to the home of the deceased and by just sparsely looking at the corpse decide that he is convinced of a natural death.

In Belgium, the physician performing the (external) post-mortem examination has to state on the official death certificate whether there are ‘medico-legal objections for cremation or burial’. This would be the case if the death is ‘certainly or suspected to be due to an external cause’. In the concept of ‘certainly or suspected to be’ there is ample room for diverse interpretations of the terms. Admittedly ‘certainly’ sounds reasonably clear and seems to adhere to the legal terms ‘probability verging on certainty’, ‘moral certainty’ and ‘beyond doubt’.

In Germany, a physician needs to state after (external) post-mortem examination whether a death is natural, unnatural or ‘unknown/unexplained’. There is no mention of any degree of certainty in German law, but the possibility to choose 'unknown/unexplained' gives the physician the opportunity to utter any doubts they might have.

In England, the degree of certainty is not on the natural side of the death spectrum but on the unnatural/non-natural side of the spectrum. A death should be reported to the coroner when a doctor knows, or has reasonable cause to suspect, that the death falls under one of the types of death that are reportable to the coroner. A reportable death is a violent or unnatural death, a death with an unknown cause or a death in custody or otherwise in state detention, even if of natural cause. ‘Reasonable cause to suspect’ means, according to Dorries, that the doctor must have formed a genuine suspicion, based on an objective assessment, upon something of substance [5].

## Criteria

Facts and circumstances play a role in the decision made by the physician or the coroner about natural or unnatural death. The question remains on which criteria or facts and circumstances the conviction of a natural or unnatural death is based upon. Criteria that appear to be of importance are time between an occurrence and death, age of the deceased, and who or what is responsible for the death.

### Time and age

When considering the age of the deceased person, it is clear that a person of old age has a far greater chance of dying than a younger person. For example, in the Netherlands, about 152,000 people a year die, of whom only 1046 are under the age of 20, i.e. only 0.69% of all the deceased were under the age of 20 in 2018. Most people die of a disease and the susceptibility to illness grows with age. The younger the deceased the more suspicious it is that the person died at all, except of course when diseases play a role. Thus, one would be less convinced of a natural death if the deceased is (very) young. But how young is young? Or how old is old? One’s own age, i.e. the age of the attending physician, will invariably play a role in this assessment. An older physician in good health, with healthy friends and family surrounding him, might be less likely to assume a natural death solely based on the fact that the deceased is a bit older.

Time is of importance in the chain of events. Consider a person who has had a severe scooter accident at 16 and becomes wheelchair-bound for the rest of his life due to paraplegia. At the age of 45, they ultimately die from the umpteenth untreatable bladder and kidney infection. Though dying from an infection is considered natural, most people do not have bladder and kidney infections that often and that severe, let alone die from them. One could pose that if this person would not have had the accident at the age of 16, he probably would not have died at the age of 45. One could also wonder how certain the doctor should be that the infection is due to the paraplegia. Or should one state that everything in life is a consequence of what happened before and this would mean that almost every death is unnatural. How far back in time does one go on the chain itself? For example, an elderly person dies of pneumonia (a natural cause of death) after spending time in the hospital (considered natural or unnatural cause of death, depending on the country) after having undergone surgery (considered natural or unnatural cause of death, depending on the country) due to a broken hip after a fall (an unnatural cause of death) caused by her Parkinsons (a natural cause of death). Is the conclusion ‘unnatural’ if something unnatural occurs in the chain of events? Or should one conclude that an illness was at the base of the event? In most, if not all, cases one will always find something that may be interpreted as an external cause and leads to a conclusion of unnatural death.

Another point of discussion would be the quantity of substance administered over time. This appears to be decisive for the question whether death is natural or unnatural. For example, at least in the Netherlands, acute alcohol intoxication resulting in death is viewed as an unnatural death, but dying from chronic alcohol abuse is considered a natural death. In the same line of thought, what should we think of dying off lung cancer after years of smoking or from asbestos exposure?

Though lung cancer could be considered as a natural death, one could argue that smoking and asbestos as causes of the lung cancer are external causes and thus could be considered an unnatural cause of death. Strangely, the discussion about natural or unnatural does not occur after smoking, but it does after asbestos-exposure. This might have to do with the odds of a disease occurring when exposed to the substance. But also with the question of responsibility or liability or even provability and causality.

### Who/what is responsible for the death?

#### Person themselves

When considering death caused by the person themselves, the first thing that comes to mind is suicide. This will be considered as an unnatural death. But if the person suffered depression for many years with suicidal thoughts most of the time, couldn't suicide be seen as a natural progression of the disease? Regardless of the fact that the person may or may not have sought help, treatment may not have been successful. To compare this, for example, with people suffering from cancer, some will seek help and treatment, but perhaps to no avail. Others may refuse treatment and will eventually also die of cancer. In these cases, refusing help is not considered to be suicide. If a person is very ill and cannot drink or eat anymore, doctors, family and patient may decide that it is for the best and leave it at that, besides making sure the patient is comfortable and not suffering. This will be considered as a natural death. And what if a person of very old age, but seemingly relatively healthy, decides it has been a wonderful life but does not want to continue on living with the risks of getting less healthy, more dependent, having already lost all their friends, and thus decides to stop eating and drinking? Is this considered suicide or could it be considered old age? The Dutch guidelines on the matter advise the involved physician to consider this a natural death [6].

Another issue to consider is the lifestyle of the person. In this day and age, we all know that smoking and drinking excessively is bad for your health. We all know that fatty foods are unhealthy and that we should exercise more. If a person is knowingly obese, smokes and drinks, and dies of cardiac problems due to this unhealthy lifestyle, the question might arise whether or not this could be considered as an unnatural death. Unnatural because they could be to blame, thus a weird kind of ‘long term’ suicide or unnatural because smoking and drinking and unhealthy food can be considered external factors. But ultimately in these cases, the physicians in all the examined countries will consider this a natural death.

#### Caused by another person

Death brought upon by another person will quickly be viewed as unnatural. Especially if it is done on purpose, as for instance in cases of manslaughter or murder. But how much morphine does it take to tip the balance from pain relief to deathly sedation in a frail person? Everything is done on purpose and arguably in the best interest of the patient, but is it a natural cause of death? In the Netherlands, death after taking palliative measurements is perceived as a natural death. Death after euthanasia or assisted suicide is considered to be an unnatural death [7].

Even more complicated are the situations in which something is not done, which could have been done, whether omission or negligence. A person comes in with non-specific symptoms and eventually dies of pancreatitis that has not been recognised. Could this be considered as unnatural? Or an elderly patient calls her GP with symptoms of pneumonia and the doctor fails to recognise the urgency. If the patient dies of her pneumonia, in view of her age and already frail health, is there a question of neglect and thus an unnatural death? Or when a patient dies during or after a rather complicated operation. Where does a medical complication cross over into a medical error/negligence? So far in the Netherlands, these cases are considered as natural death, unless obvious mistakes were made. In England and Wales, medical complications are regarded as unnatural causes of death and have to be reported to the coroner.

Another example could be the discussion around vaccinations. A parent or parents may wilfully and explicitly deny their child vaccinations because of a myriad of reasons. But if the child contracts a disease for which it could have been vaccinated and dies from that disease, will that be considered a natural or an unnatural cause of death? Does the fact that it could have been prevented play a role in this decision? Similarly, if the mother does not use folic acid during pregnancy and the child dies of the consequences of a neural tube defect, is this a natural or unnatural cause of death? Or if the mother does not comply with the dietary restriction during pregnancy or uses drugs and alcohol in excess during pregnancy and the child dies of the consequences? In the Netherlands again, these cases will be viewed as natural deaths. As they will be in England and Wales. The amount of risk of a disease or infection does strangely not affect this conclusion. But if a parent would administer their child a substance of which it is known that the chance of getting ill and dying is high, this would be considered unnatural.

Without going into details about provability of consequences, how would a death by heart attack be considered, if the said heart attack was brought upon by an assault on the person [8]? One could at the very least doubt the natural death.

#### Another living organism

Death brought upon by another living organism may not be so clearly categorised into a natural or unnatural cause of death either. Most people would agree that being mauled to death by a bear or a dog is an unnatural cause of death. Being bitten by a snake is surely an unnatural death. But if a person comes to die after being stung by a bee or a wasp? Some would argue that the person died of an anaphylactic shock and thus the death may be considered natural. But when one looks at the definition of unnatural cause of death from a medical point of view, it is stated that an unnatural cause of death is, amongst other things, a death due to an external cause. A bee sting is very much an external cause or external factor. But the same could actually be argued about bacteria and viruses. If death is caused by a viral or bacterial infection, then it is certainly viewed as a natural cause of death. So what is the criterion used to determine whether it is a natural cause of death or an unnatural cause of death? Is it a matter of size? But where, then, is the tipping point? How big or small does the living organism have to be to fall in one category or the other? It seems rather arbitrary to state that death by bee is natural but death by snake is unnatural as both inject poison. Does the criterion lay in the distinction that death by a bear or a venomous snake is from the violent act itself or from the poison and death by a bee is a physical reaction to a certain form of nonlethal venom, even if both the animal and the insect do what naturally comes to them?

Or should one also consider the fact that a living organism could be a vector for another living organism such as a bacterium or a virus? For example dying from malaria after being stung by a mosquito, secondary death by the Zika virus, or dying from the Marburg virus after exposure to infected bats. In what category would the death caused by the infection after a dog-bite fall?

And what about the ability to use bacteria or viruses as weapons? Dying from HIV or AIDS is considered a natural cause of death, despite the risky or intentional behaviour that may have led to the contraction of HIV. There has been a case in the Netherlands where two HIV-positive men would give sexual parties for homosexual men. The hosts would drug their guests to then inject them with their own (the perpetrators) infected blood [9]. Furthermore, it is known that there are communities that enjoy infecting healthy people and purposefully omit to disclose that they are carrying HIV [10]. Would this be considered a natural or unnatural cause of death?

#### Nature and environment

Nature is defined by ground, water, air and everything on or in it, as far as not produced by man. Being born is considered to be one of the most natural facts of life, but is surrounded by a lot of ‘unnatural behaviour’ like medical guidance and obstetric actions. Being born is hazardous and may lead to death. When this occurs, death is called natural even if a certain amount of physical violence was needed to be born. When the same sort and amount of physical violence would be expressed upon a newborn and death would occur, this death would certainly be labelled as unnatural. When it comes to death around a birth the criterion may not be the sort or amount of violence, but the way the woman in labour is assisted in that particular case.

Eating and drinking is essential to stay alive. But eating and drinking poison is considered to cause an unnatural death. The poisonousness of substances depends upon the substance, the quantity administered over time and the reaction of the receiver. To put it in the words of Paracelsus (German-Swiss physician, 1493–1541): “Everything is poison when not used by the right person, in the right way or in the right quantity”. Arsenic is poisonous in small quantities and death caused by oral intake of this substance is considered to be unnatural. Death due to the intake of a substance to which a person is allergic is considered to be natural. Death by intake of large amounts of water by a person with renal failure might be a natural death, but one could wonder if it is still so if done on purpose by the person himself.

Death brought upon by external factors of nature as for example by lightening, a tsunami or an earthquake falls in the category of unnatural cause of death, regardless of the jurisdiction or country. Death caused by pollution will have to take into consideration the time it might take to die from said pollution and thus the provable causality. Death caused by Ebola is natural. One could wonder why. If a whole village is decimated by the Ebola virus, for example, it will in all likelihood be viewed as natural cause of death, but if the same village disappears in an earthquake and everybody died, it will be most probably viewed as an unnatural cause of death.

Radiation is a natural and an unnatural factor. Death by radiation from polonium administered by serving a cup of tea is considered unnatural (e.g. the poisoning of ex-KGB spy Alexander Litvinenko), even though the polonium might be naturally found. Death after a nuclear event like that occurred in Chernobyl is an unnatural death. But the question arises when considering the death, usually by cancer, of the second generation after Chernobyl.

## Conclusion

The terms natural and unnatural/nonnatural death are not clearly defined and give rise to the discussion as seen in this article. Not only are the terms variably used between medical professions but they also give rise to the discussion within the different medical professions. Furthermore, the conclusion of a natural and unnatural/nonnatural death is based on conviction, which itself is apparently often based on very little investigation. The boundaries of the terms are unclear, but a physician is forced to make a dichotomous decision, natural or unnatural/nonnatural death. This dichotomous medical conclusion has important consequences in the judicial system and leads to a decision on whether or not the death is investigated further.

If we want to comply with Article 2 ECHR, we need a system in which the circumstances surrounding and leading up to a death are properly investigated and established. This should lead to a report to an independent legal expert, who is able to decide if and what conclusion can be drawn, from a judicial and a public point of view. Thereby, making the distinction and the use of the terms natural and unnatural/nonnatural obsolete.

## Abbreviations

Not applicable.

## Data Availability

Not applicable.
